# Inebilizumab in AQP4‐Seropositive NMOSD: One‐Year Follow‐Up From a Multicenter, Real‐World Study

**DOI:** 10.1002/acn3.70512

**Published:** 2026-08-23

**Authors:** Mengcui Gui, Jiayang Zhan, Wei Li, Tao Jin, Dan He, Daishi Tian, Zhijun Li, Jing Lin, Yue Li, Hongyu Zhou, Bitao Bu

**Affiliations:** ^1^ Department of Neurology Tongji Hospital, Tongji Medical College, Huazhong University of Science and Technology Wuhan Hubei China; ^2^ Hubei Key Laboratory of Neural Injury and Functional Reconstruction Huazhong University of Science and Technology Wuhan Hubei China; ^3^ Department of Neurology Henan Provincial People's Hospital, Zhengzhou University People's Hospital Zhengzhou Henan China; ^4^ Department of Neurology, Neuroscience Center The First Hospital of Jilin University Changchun Jilin China; ^5^ Department of Neurology Zhongnan Hospital of Wuhan University Wuhan Hubei China; ^6^ Department of Neurology West China Hospital, Sichuan University Chengdu Sichuan China

**Keywords:** aquaporin‐4, inebilizumab, neuromyelitis optica spectrum disorder, real‐world evidence

## Abstract

**Objective:**

Real‐world evidence on inebilizumab among neuromyelitis optica spectrum disorder (NMOSD) patients is lacking. This study assessed inebilizumab among Chinese patients with aquaporin 4 autoantibody (AQP4‐IgG)‐seropositive NMOSD in a real‐world setting.

**Methods:**

This multicenter, prospective, observational study enrolled patients with AQP4‐IgG‐seropositive NMOSD who received at least one dose of inebilizumab. The primary outcome was the time to first adjudicated NMOSD attack.

**Results:**

A total of 143 patients with AQP4‐IgG‐seropositive NMOSD were included. Over a median follow‐up of 12.4 months (range: 0.4–25.4), five patients (3.50%) experienced attacks, with a 1‐year cumulative incidence of 4.54% (95% confidence interval, 1.66–9.70). Annualized attack rate decreased from 1.02 to 0.03 after inebilizumab treatment, and the Expanded Disability Status Scale scores were significantly improved, with a median change of −0.50 (range, −5.0 to 1.5; *p* < 0.0001; worsening rate, 0.70%) at 1 year. The most common treatment‐emergent adverse events were urinary tract infection (6.29%), and one case of pneumonia (0.70%) was reported as serious adverse event. B‐cell depletion was effectively achieved, with only 1 patient reporting hypogammaglobulinemia.

**Conclusion:**

Inebilizumab demonstrated real‐world effectiveness and a manageable safety profile in a diverse population of patients with AQP4‐IgG‐seropositive NMOSD, supporting the findings of the pivotal N‐MOmentum trial.

## 1 Introduction

Neuromyelitis optica spectrum disorder (NMOSD) is a rare, relapsing inflammatory disease of the central nervous system (CNS) characterized by core clinical manifestations, including optic neuritis, myelitis, and brain or brainstem syndromes, with most cases seropositive for aquaporin 4 (AQP4) autoantibodies (AQP4‐IgGs) [1, 2]. The worldwide prevalence rates of NMOSD range from approximately 0.07 to 10 per 100,000 people, and incidence rates vary from 0.029 to 0.880 per 100,000 population annually [3], which vary by ethnicity and geography [2, 4, 5]. The treatments for acute NMOSD attacks include intravenous methylprednisolone, intravenous immunoglobulin (IVIg), and plasmapheresis [6, 7, 8]. Nevertheless, NMOSD typically follows a relapsing course in 80%–90% of patients [1], underscoring the importance of attack prevention, as accumulating damage from attacks contributes to progressive disability.

B cells play a central and multifaceted role in the pathogenesis of NMOSD, through the production of pathogenic autoantibodies targeting AQP4, cytokine secretion, antigen presentation, and defective immune regulation [9, 10, 11]. Maintenance therapy in NMOSD has rapidly evolved toward targeted biologics, including B‐cell targeting antibodies. Inebilizumab is a humanized, affinity‐enhanced, afucosylated IgG1 kappa monoclonal antibody that targets the B‐cell surface antigen CD19. In the N‐MOmentum study, inebilizumab significantly lowered the risk of NMOSD attacks compared with placebo, particularly in AQP4‐IgG‐seropositive patients [12]. At the end of the 4‐year follow‐up, sustained clinical benefits were observed with annualized attack rates decreased year‐on‐year [13]. Thus, while CD20 depletion with rituximab is approved for NMOSD in Japan [14], CD19 depletion with inebilizumab remains the mainstay in maintenance care for NMOSD in most countries and regions worldwide.

Although the pivotal, randomized N‐MOmentum study has confirmed the efficacy and safety of inebilizumab for the treatment of NMOSD, patients encountered in clinical practice, who may not conform to the stringent eligibility criteria, suggest the persistent heterogeneity between trial and real‐world settings. In addition, the treatment patterns and adherence often differ considerably across countries and regions, and may not align with those reported in trials. Hence, real‐world evidence is necessary to assess the approved drugs in routine clinical practice. Notably, with the racial variations in NMOSD manifestations highlighted by previous studies [15, 16], the N‐MOmentum study included only about 20% of participants of Asian descent, and did not recruit patients from mainland China [12]. To fill this knowledge gap, the present real‐world study aimed to evaluate the effectiveness and safety of inebilizumab among Chinese patients with AQP4‐IgG‐seropositive NMOSD.

## 2 Methods

### 2.1 Study Design and Patients

This multicenter, prospective, observational study was conducted across five tertiary hospitals in Central, Western, and Northeastern China.

Eligible patients aged 18 years or older, with a confirmed diagnosis of AQP4‐IgG‐seropositive NMOSD according to the 2015 International Panel for NMO Diagnosis (IPND) criteria [17], were enrolled, both newly diagnosed and relapsing individuals. Patients were eligible if they were indicated for inebilizumab treatment or had received inebilizumab with at least one follow‐up visit after inebilizumab therapy. AQP4‐IgG positivity was defined as detectable AQP4‐IgG in serum and/or cerebrospinal fluid, or a documented history of a prior AQP4‐IgG‐seropositive record. The exclusion criteria included the presence of clinically significant systemic or hematologic disorders, any acute infection requiring treatment within 4 weeks before screening, chronic infections necessitating ongoing treatment, active hepatitis, human immunodeficiency virus (HIV) infection, malignancy, pregnancy or breastfeeding, substance or alcohol abuse or dependence, or known drug or allergy history.

### 2.2 Standard Protocol Approvals, Registrations, and Patient Consents

This study was in accordance with the principles of the Declaration of Helsinki and adhered to the International Conference on Harmonization Good Clinical Practice guidelines. The research protocol, including all procedures involving human participants, was reviewed and approved by the following institutional review boards (IRBs) or independent ethics committees (ECs): the Medical Ethics Committee of Tongji Medical College, Huazhong University of Science and Technology (Approval No. TJ‐IRB202401054), the Ethics Committee of West China Hospital, Sichuan University (Approval No. 2024‐1676), the Ethics Committee of the First Hospital of Jilin University (Approval No. 24K218‐001), the Medical Ethics Committee of Zhongnan Hospital of Wuhan University (Approval No. 2024224K), and the Medical Ethics Committee of Henan Provincial People's Hospital, Zhengzhou University People's Hospital (Approval No. 2024‐155). Written informed consent was obtained from all patients.

### 2.3 Data Collection and Follow‐Up

Demographic, clinical, radiological, and immunological information before initiating inebilizumab treatment was collected, including sex, age, family history, disease course, details of previous attacks, Expanded Disability Status Scale (EDSS) scores, AQP4 antibody titer, laboratory test, and imaging. Treatment was determined by treating physicians based on patients' condition and was not prespecified in the study protocol. In the label, inebilizumab was administered at 300 mg on Day 1 and Day 15, followed by a maintenance dose of 300 mg every 6 months.

Patients were followed up on Day 1, Day 15, and every 3 months for 2 years to document attacks, medication use, and adverse events (AEs) on‐site or by phone.

### 2.4 Outcomes and Assessments

The primary outcome was the time to first adjudicated NMOSD attack, defined as the duration from Day 1 to the onset of an NMOSD attack as determined by the investigator. An attack was characterized by the emergence of new neurological symptoms or the worsening of existing NMOSD‐related symptoms lasting for more than 24 h, occurring at least 30 days after a previous attack. Secondary outcomes included the annualized attack rate (AAR), changes in EDSS scores from baseline, proportion of patients with worsening EDSS at 6 months and 12 months, and treatment patterns. EDSS worsening was defined as an increase of ≥ 2.0 points for baseline EDSS of 0, ≥ 1.0 points for baseline EDSS between 1.0 and 5.0, and ≥ 0.5 points for baseline EDSS ≥ 5.5 [12]. The analysis of treatment pattern included concomitant treatment, treatment compliance of inebilizumab (defined as the actual number of infusions/theoretical number of infusions), treatment coverage day ratio (defined as treatment‐covered days/actual follow‐up days; for patients who did not receive the Day 15 treatment, the Day 1 dose was considered to cover 91 days), and treatment duration between inebilizumab administrations.

Exploratory outcomes were the cumulative total of active magnetic resonance imaging (MRI) lesions (defined as new gadolinium‐enhancing or new/enlarging T2 lesions across the optic nerve, brain, brainstem, and spinal cord), changes from baseline in anti‐AQP4 antibody titers, B‐lymphocyte subsets, and immunoglobulin levels. Serum AQP4 antibody titers and seroconversion were assessed using a cell‐based quantitative assay (CBA), while B‐cell subsets and cytokines were analyzed by flow cytometry. All AEs and serious AEs (SAEs) were documented throughout the study. Hypogammaglobulinemia was operationally defined as serum IgG < 4 g/L, in line with published study [18, 19].

### 2.5 Statistical Analysis

Continuous variables were presented as mean ± standard deviation or median with range, while categorical variables were expressed as frequencies and percentages. The rates were presented along with 95% confidence intervals (CIs) calculated using the Clopper–Pearson exact method. The time to first NMOSD attack was evaluated using the Kaplan–Meier survival analysis, from which the median time, 95% CI, and cumulative attack rate were obtained. Differences in attack rates and EDSS scores before and after treatment were assessed using the Wilcoxon signed‐rank test. Additional subgroup analyses were performed in patients with prior rituximab use, and in other prespecified subgroups. All statistical tests were two‐sided, and a *p*‐value of < 0.05 was considered statistically significant. Statistical analyses were performed using SAS 9.4 software (SAS Institute, Cary, NC, USA).

## 3 Results

### 3.1 Baseline Characteristics of the Study Population Before Inebilizumab Treatment

Between September 19, 2024, and June 30, 2025, 143 patients with AQP4‐IgG‐seropositive NMOSD were included. The average age of included patients before inebilizumab treatment was 45.0 ± 13.72 years, and 85.31% were female. The median disease duration before inebilizumab treatment was 1.51 years (range, 0.00–26.43). Nearly all patients (96.50%, *n* = 138) had received prior treatment, and 75.52% (*n* = 108) had undergone maintenance therapy before inebilizumab treatment. The most common maintenance agents were mycophenolate mofetil (MMF) (22.38%, *n* = 32), tacrolimus (16.78%, *n* = 24), and rituximab (12.59%, *n* = 18), followed by other non‐biological or biological immunosuppressants. Twenty‐seven patients had comorbid autoimmune diseases, most commonly thyroid disease (9.09%, *n* = 13). There were 29 (20.28%) patients with positive hepatitis B core antibodies and negative hepatitis B surface antigens, among whom 2 received entecavir. Five (3.50%) patients had latent tuberculosis infection diagnosed by T‐SPOT, among whom 2 received rifampin and isoniazid. The median baseline EDSS score was 3.0 (range, 0.0–9.0). The demographic and clinical characteristics of the study population at the time of inebilizumab administration are shown in Table 1.

**TABLE 1:** Baseline characteristics of the patients before inebilizumab treatment.

	Total (*N* = 143)
Age, years, mean ± SD	45.0 ± 13.72
Gender	
Male	21 (14.69)
Female	122 (85.31)
Disease duration, year	1.51 (0.00, 26.43)
Type of last relapse, *n* (%)	
Optic neuritis	32 (22.38)
Acute myelitis	57 (39.86)
Optic neuritis and acute myelitis	34 (23.78)
Others	20 (13.99)
Baseline EDSS score	3.0 (0.0, 9.0)
Comorbid autoimmune disease	
Sjögren's syndrome	7 (4.90)
Thyroid disease	13 (9.09)
Myasthenia gravis	1 (0.70)
Autoimmune hepatitis	1 (0.70)
Others	6 (4.20)
Previous treatment	
Any treatment	138 (96.50)
Plasmapheresis	6 (4.20)
IVIg	24 (16.78)
Previous maintenance treatment	
Any maintenance treatment	108 (75.52)
MMF	32 (22.38)
Azathioprine	10 (6.99)
Tacrolimus	24 (16.78)
Cyclophosphamide	2 (1.40)
Other non‐biological immunosuppressants	3 (2.10)
Ofatumumab	3 (2.10)
Rituximab	18 (12.59)
Telitacicept	2 (1.40)
Eculizumab	1 (0.70)

Abbreviations: EDSS, Expanded Disability Status Scale; IVIg, intravenous immunoglobulin; MMF, mycophenolate mofetil; SD, standard deviation.

In a subset of 62 patients with prior maintenance therapy and detailed documentation on the reasons for initiating inebilizumab, the most common reasons were relapse or inadequate disease control on prior therapy (*n* = 35, 56.45%), patient preference (*n* = 21, 33.87%) and adverse events or tolerability issues (*n* = 6, 9.68%). Among the 17 patients with prior rituximab use and available data, the distribution of reasons was similar to that in the overall cohort, with the most common reasons being patient preference (*n* = 8, 47.06%), relapse or inadequate disease control on prior therapy (*n* = 7, 41.18%) and adverse events or tolerability issues (*n* = 2, 11.76%).

### 3.2 Treatment Pattern

As of August 20, 2025, the median number of inebilizumab infusions was 4 (range, 1–5), with 139 patients (97.20%) completing the Day 1 and Day 15 inebilizumab treatments. Among the 120 patients who received a third dose, the median interval between the second and third dose was 178 days (IQR, 167, 184; range, 137–320). Among patients who received a fourth dose (*n* = 82) and a fifth dose (*n* = 41), the median interval was 184 days (IQR, 181, 192; range, 170–247) and 183 days (IQR, 181, 188; range, 171–228), respectively. The proportion of patients with a treatment compliance of ≥ 80% was 95.10% (*n* = 136), and the proportion of patients with a treatment coverage day ratio of ≥ 80% was 93.71% (*n* = 134).

The median duration of concomitant corticosteroid therapy during inebilizumab treatment was 6.16 months (range, 0.03–23.79), with the proportions of patients with concomitant corticosteroids of 45.45% (65/143), 43.2% (54/125), and 20.93% (18/86) at 3, 6, and 12 months after initiating inebilizumab, respectively. The median duration of concomitant immunosuppressant therapy was 6.51 months (range, 0.20–18.01). The proportions of patients receiving concomitant treatment at 3, 6, and 12 months were 5.59% (8/143), 5.60% (7/125), and 1.16% (1/86) for MMF, 1.40% (2/143), 1.60% (2/125), and 1.16% (1/86) for azathioprine, and 6.29% (9/143), 6.40% (8/125), and 2.33% (2/86) for tacrolimus.

By the data cut‐off date, three patients had discontinued inebilizumab, defined as cessation of treatment with subsequent switch to another maintenance therapy. Two patients discontinued due to adjudicated relapses and changed to alternative long‐term immunosuppressive regimens. The third discontinuation occurred in a patient with progressively declining immunoglobulin levels and B‐cell counts, in whom the treating physician judged ongoing inebilizumab therapy to be inappropriate.

### 3.3 Primary and Secondary Outcomes

**FIGURE 1:**
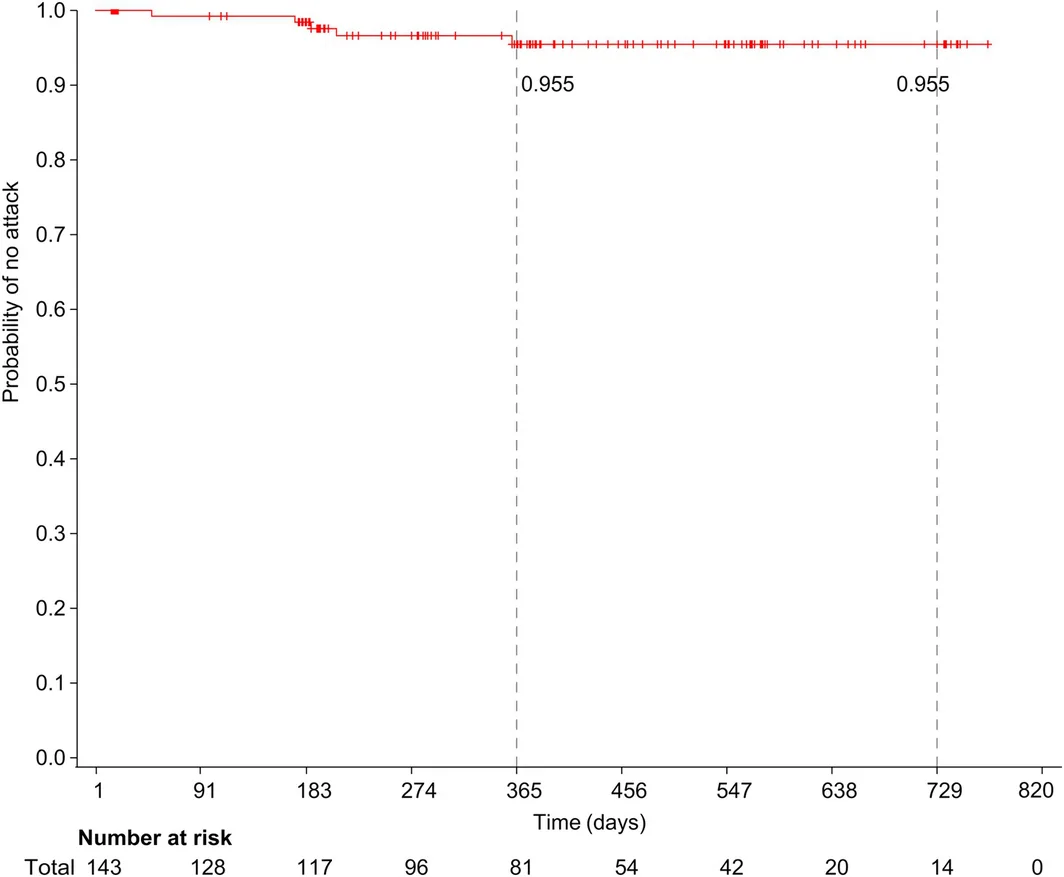
First adjudicated attack of neuromyelitis optica spectrum disorder in all patients.

As of August 20, 2025, the median follow‐up time was 12.4 months (range, 0.4–25.4). Among the 143 patients, five (3.50%) experienced an attack, with the median time to first adjudicated attack not reached (Figure 1). The 1‐year cumulative incidence of attack was 4.54% (95% CI, 1.66–9.70), and the probability of being attack‐free was 95.46% (95% CI, 89.33–98.11) (Table 2). Among 27 patients with comorbid autoimmune diseases, 2 (7.41%) experienced an NMOSD attack, with a 1‐year cumulative incidence of attack of 9.66% (95% CI, 1.52–27.13). For the 18 patients with prior rituximab use, 1 (5.56%) experienced an NMOSD attack, with a 1‐year cumulative incidence of attack of 5.88% (95% CI, 0.35–24.21). Among the 62 patients with documented reasons for initiating inebilizumab, 35 patients initiated inebilizumab due to relapse or inadequate disease control on prior therapy, of whom 3 (8.57%) experienced an NMOSD attack, yielding a 1‐year cumulative incidence of attack of 9.14% (95% CI, 2.26 to 22.06). Among the remaining 27 patients who initiated inebilizumab for other reasons, none experienced an NMOSD attack.

**TABLE 2:** Primary, secondary, and safety outcomes.

	Total (*N* = 143)
**Primary outcome: First adjudicated attack**	
Events, *n* (%)	5 (3.50)
Median time to first adjudicated relapse, weeks (95% CI)	Not reached
1‐year cumulative attack rate, % (95% CI)	4.54 (1.66, 9.70)
1‐year cumulative attack‐free rate, % (95% CI)	95.46 (89.33, 98.11)
**Secondary outcomes**	
AAR, (95% CI)	
Before inebilizumab treatment	1.02 (0.89, 1.17)
After inebilizumab treatment	0.03 (0.01, 0.08)
EDSS score, median (range)	
Before inebilizumab treatment	3.00 (0.0, 9.0)
Change from baseline at 6 months after inebilizumab treatment	−0.50 (−5.0, 0.0)
*p* value comparing to baseline	< 0.0001
EDSS worsening rate, % (95% CI)	0.00 (0.00, 2.55)
Change from baseline at 1 year after inebilizumab treatment	−0.50 (5.0, 1.5)
*p* value comparing to baseline	< 0.0001
EDSS worsening rate, %, (95% CI)	0.70 (0.02, 3.83)
**Exploratory outcomes**	
MRI lesion	
Events, *n* (%)	4 (2.80)
Incidence, % (95% CI)	2.80 (0.77, 7.01)
AQP4‐IgG titer, median (range)	
Before inebilizumab treatment [*n* = 117]	2.00 (0.00, 3.51)
Change from baseline at 6 months after inebilizumab treatment [*n* = 16]	0.00 (−2.00, 1.51)
*p* value	0.8633
AQP4‐IgG seronegative, *n* (%)	3 (2.10)
Change from baseline at 1 year after inebilizumab treatment [*n* = 11]	0.00 (−2.00, 1.49)
*p* value	0.7500
AQP4‐IgG‐seropositive, *n* (%)	3 (2.10)

Abbreviations: AQP4‐IgG, aquaporin autoantibodies; CI, confidence interval; EDSS, Expanded Disability Status Scale; MRI, magnetic resonance imaging.

Treatment with inebilizumab was associated with a substantial reduction in AAR, with the AAR of 1.02 (95% CI, 0.89–1.17) before treatment, and decreased markedly to 0.03 (95% CI, 0.01–0.08) after treatment (Table 2). Among patients who had previously received maintenance therapy, the median AAR before treatment was 1.50 (range, 0.00–19.23), which decreased significantly to 0.00 (range, 0.00–3.48) after treatment with inebilizumab (median change from baseline, −1.39; range, −19.23 to 0.62; *p* < 0.0001).

After treatment with inebilizumab, there was a statistically significant improvement in EDSS score, with a median change of −0.50 (range, −5.0 to 0.0; *p* < 0.0001) at 6 months and −0.50 (range, −5.0 to 1.5; *p* < 0.0001) at 1 year, respectively. The EDSS worsening rate was 0.00% (95% CI, 0.00–2.55) at 6 months and 0.70% (95% CI, 0.02–3.83) at 1 year (Table 2).

### 3.4 Exploratory Outcomes

**FIGURE 2:**
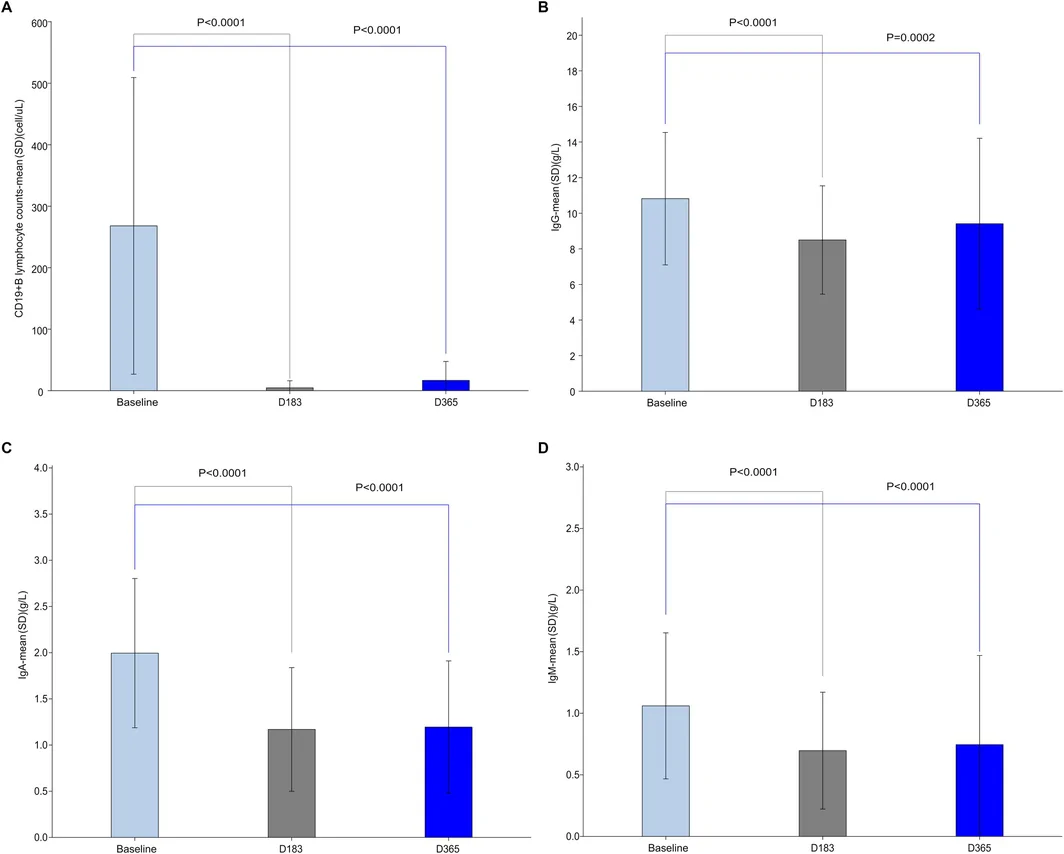
(A) CD19+ B lymphocytes counts and (B) IgG, (C) IgA, and (D) IgM levels before and after inebilizumab treatment.

One year after initiating inebilizumab therapy, the cumulative incidence of active MRI lesions was low at 2.80% (*n* = 4; 95% CI, 0.77–7.01). Sixteen and eleven patients had available data for AQP4‐IgG titer at 6 months and 1 year, and three patients were seronegative for AQP4‐IgG after 6 months of inebilizumab. Significant reductions in CD19+ B‐cell counts, IgG, IgA, and IgM were observed as early as 6 months after starting inebilizumab, and counts remained lower than baseline at 1 year, with over 90% of patients maintaining IgG levels at or above 5 g/L at each visit point (Figure 2 and Table 3).

**TABLE 3:** B‐cell lymphocytes and immunoglobulin changes during the follow‐up.

	Total (*N* = 143)
B lymphocytes, cells/μL, median (range)	
Baseline [*N* = 79]	203.0 (0.0, 1103.0)
6 months after inebilizumab treatment [*N* = 83]	0.0 (0.0, 89.0)
*p* value comparing to baseline	< 0.0001
12 months after inebilizumab treatment [*N* = 57]	1.2 (0.0, 163.0)
*p* value comparing to baseline	< 0.0001
IgG, g/L, median (range)	
Baseline [*N* = 94]	10.5 (4.8, 24.9)
≥ 5 g/L, *n* (%)	92 (97.87)
6 months after inebilizumab treatment [*N* = 85]	8.2 (2.9, 21.1)
≥ 5 g/L, *n* (%)	77 (90.59)
*p* value comparing to baseline	< 0.0001
12 months after inebilizumab treatment [*N* = 53]	8.4 (2.7, 32.7)
≥ 5 g/L, *n* (%)	50 (94.34)
*p* value comparing to baseline	0.0002
IgA, g/L, median (range)	
Baseline [*N* = 91]	1.8 (0.6, 4.6)
6 months after inebilizumab treatment [*N* = 82]	1.1 (0.1, 3.5)
*p* value comparing to baseline	< 0.0001
12 months after inebilizumab treatment [*N* = 53]	1.1 (0.1, 3.5)
*p* value comparing to baseline	< 0.0001
IgM, g/L, median (range)	
Baseline [*N* = 92]	0.9 (0.2, 3.0)
6 months after inebilizumab treatment [*N* = 83]	0.6 (0.1, 2.4)
*p* value comparing to baseline	< 0.0001
12 months after inebilizumab treatment [*N* = 53]	0.6 (0.0, 4.7)
*p* value comparing to baseline	< 0.0001

### 3.5 Safety

In this study, the incidence of treatment‐emergent AEs (TEAEs) was 32.87% (*n* = 47), while SAEs occurred in 0.70% of cases, corresponding to one case of pneumonia. The most common TEAEs (occurring in at least 2.5% of patients) were urinary tract infection (6.29%, *n* = 9), hyperlipidemia (4.90%, *n* = 7), anemia (2.80%, *n* = 4), kidney dysfunction (2.80%, *n* = 4), fever (2.80%, *n* = 4), hypoproteinemia (2.80%, *n* = 4), and leukocytosis (2.80%, *n* = 4) (Table 4). Hypogammaglobulinemia was reported in one patient, and no infusion‐related reaction was documented.

**TABLE 4:** Summary of adverse events.

	All (*N* = 143)
Any TEAE	47 (32.87)
Any SAE	1 (0.70)
The most common TEAE (≥ 2.5%)	
Urinary tract infection	9 (6.29)
Hyperlipidemia	7 (4.90)
Anemia	4 (2.80)
Hypoproteinemia	4 (2.80)
Kidney dysfunction	4 (2.80)
Leukocytosis	4 (2.80)
Fever	4 (2.80)

Abbreviations: SAE, serious adverse event; TEAE, treatment‐emergent adverse event.

## 4 Discussion

This multicenter, prospective, observational study evaluated the real‐world effectiveness and safety of inebilizumab in 143 Chinese patients with AQP4‐IgG‐seropositive NMOSD and reported a 1‐year cumulative attack rate of 4.54% and an attack‐free rate of 95.46%, with markedly decreased AAR after inebilizumab treatment. The EDSS score significantly improved with inebilizumab administration, with active MRI lesions observed in only 2.80% of patients at 1 year. Three patients were seronegative for AQP4‐IgG after 6 months, and the monitoring of B‐cell lymphocyte counts and immunoglobulin levels indicated effective B‐cell depletion, with only 1 patient reporting hypogammaglobulinemia. Overall, this study provided evidence on the real‐world effectiveness and a manageable safety profile of inebilizumab in a diverse population with AQP4‐IgG‐seropositive NMOSD. To the best of the authors' knowledge, this study represented the largest real‐world study of inebilizumab in NMOSD to date, as well as the study with the largest Chinese population using inebilizumab.

In the N‐MOmentum study, inebilizumab achieved an attack‐free rate of 87.6% and an EDSS worsening rate of 16% among AQP4‐IgG‐seropositive NMOSD patients at 6 months [12], and an attack‐free rate of 85% with an AAR of 0.185 at 1 year after treatment [13]. In comparison, this study demonstrated an attack‐free rate of 95.46% at 1 year, with an AAR of 0.03 and an EDSS worsening rate of 0.70%, which aligns with findings from another real‐world cohort of 22 NMOSD patients treated with inebilizumab [20]. Beyond effective control in disease relapse, exploratory analyses in this study further confirmed the durable depletion of B cells. It should be noted that the inclusion of 143 Chinese NMOSD patients in the present analysis adds valuable insights into treatment outcomes in this demographic, considering racial variations in NMOSD manifestations [15, 16], and only approximately 20% of participants were of Asian descent in the N‐MOmentum study [12]. In addition, plasmapheresis was used by 36% of participants for acute attack management in the N‐MOmentum study [12], whereas only 4.20% of patients in the present cohort had previously undergone this procedure, likely reflecting differences in healthcare resource availability. Notably, azathioprine was the most common non‐biologic immunosuppressant for maintenance therapy in the N‐MOmentum study (39%) [12], compared with MMF, which was the leading agent in the study. Unlike the N‐MOmentum study, which excluded patients with prior rituximab exposure within 6 months or eculizumab exposure within 3 months, this study did not restrict the prior treatment history to mirror real‐world practice. These disparities in race or clinical practice might contribute to the comparatively favorable clinical outcomes in this study, relative to the pivotal N‐MOmentum study, which is a common phenomenon as evidenced by trials and real‐world studies of other biologics for NMOSD [21, 22]. Furthermore, although caution must be taken when interpreting results across studies, the therapeutic benefit observed with inebilizumab in this study appears superior compared with those reported in Chinese NMOSD patients who received non‐biologic immunosuppressants (e.g., azathioprine or MMF), with a 1‐year attack‐free rate typically ranging from 60% to 75% [23]. Therefore, the present real‐world study complemented real‐world evidence to support inebilizumab as standard maintenance therapy for Chinese patients with AQP4‐IgG‐seropositive NMOSD.

With regard to treatment pattern, nearly all participants completed both the Day 1 and Day 15 dosing and adhered to the recommended 6‐month treatment interval. Real‐world complexities, such as clinic scheduling, personal preferences, and intercurrent medical conditions, may contribute to infusion delays in routine practice. However, only a minority of patients in our cohort experienced substantial delays. As for concomitant medication, the median duration of corticosteroid treatment was 6.16 months, and approximately 40% of patients continued corticosteroids at 6 months, which echoes with the 2024 Expert Recommendations for the Appropriate Use of Inebilizumab in Neuromyelitis Optica Spectrum Disorder that at least 6 months of combined corticosteroid‐inebilizumab therapy is recommended [24]. By contrast, the N‐MOmentum study implemented a short‐term daily 20 mg prednisone between Days 1 and 14, tapered to Day 21 after treatment initiation [12], highlighting a major difference in corticosteroid exposure, which may have contributed to the numerically higher 1‐year attack‐free rate observed in this study compared to the N‐MOmentum study. In addition, no additional safety signals were identified with this combination strategy, and only a small proportion of patients remained on concomitant corticosteroids or conventional immunosuppressants at 12 months after inebilizumab initiation, reflecting the effectiveness of inebilizumab monotherapy and the recommendation to reduce the risks associated with combination therapy [24]. These findings underscore the necessity of defining the optimal duration of concomitant treatments, which should be verified in future studies.

The N‐MOmentum study excluded participants presenting with uncontrolled comorbid autoimmune disorders necessitating therapy with disease‐modifying or immunosuppressive agents [12], while this study did not restrict this and included 27 patients with comorbid autoimmune diseases, whose therapeutic responses were comparable to the overall study cohort. These results reinforce the practicality of using inebilizumab in NMOSD patients with concurrent autoimmune disorders, consistent with evidence from previous reports of NMOSD accompanied by myasthenia gravis [25] and by Sjogren's syndrome [26]. In addition, given the crucial contribution of B cells across multiple autoimmune pathologies, inebilizumab has exhibited notable efficacy in IgG4‐related disease [27] and generalized myasthenia gravis [28]. Thus, in patients burdened by several autoimmune comorbidities, inebilizumab may confer advantages by mitigating disease activity across multiple conditions. Furthermore, the N‐MOmentum study excluded individuals who were positive for hepatitis B surface antigen, or hepatitis B core antibody positive with hepatitis B surface antibody negative, and those with active or latent tuberculosis. Given the high prevalence of hepatitis B [29] and tuberculosis [30] in China, including among individuals with NMOSD, this study adopted less restrictive criteria, leading to the inclusion of 29 patients who were hepatitis B core antibody‐positive and five with latent tuberculosis. Consistent with prior case series findings, inebilizumab use in this subset proved to be safe, showing a minimal risk of hepatitis B reactivation or tuberculosis reactivation [31]. By including a more heterogeneous population, this study provides real‐world evidence for the effectiveness and safety of inebilizumab in a broader NMOSD population.

The safety profile of inebilizumab observed in this study was comparable to findings from both the pivotal N‐MOmentum study [12, 13] and existing real‐world study [20]. With an overall incidence of TEAEs of 32.87%, only one SAE of pneumonia was observed throughout the study period, and the most common TEAEs were urinary tract infection, which might be associated with B‐cell suppression and reduced immunologic surveillance. Importantly, no unexpected or novel safety signals emerged compared to other biologics [10, 22, 32], supporting the notion that inebilizumab exhibits a tolerable and predictable safety profile across diverse patient populations. Despite observed reductions in B‐cell lymphocytes and immunoglobulins, only one patient reported hypogammaglobulinemia. These results reinforce evidence from the N‐MOmentum study [12, 13], indicating that the treatment's benefits in relapse prevention outweigh its potential risks when used in appropriately selected patients, underscoring the continued role of inebilizumab as an effective and well‐tolerated option for NMOSD management.

This study had limitations. Firstly, the follow‐up was relatively short, and the analyzable data were limited to those that were available in the patient charts, while the missing data for the exploratory outcomes limited the conclusions that could be reached on their behalf. These exploratory assessments were not mandated by the protocol and were performed at the discretion of the treating physicians and patients. Secondly, it was a single‐group study without a comparator group. Finally, there is a potential for underreporting of attacks due to less standardized follow‐up and monitoring compared to trials, a phenomenon also observed in real‐world studies of other monoclonal antibodies [21, 22]. Additionally, the definition of relapse differed from that used in the pivotal N‐MOmentum study.

Inebilizumab demonstrated real‐world effectiveness and a manageable safety profile in a diverse population of Chinese patients with AQP4‐IgG‐seropositive NMOSD, including those with coexisting autoimmune diseases, viral hepatitis, and controlled tuberculosis. These results support the findings of the pivotal N‐MOmentum trial and further establish inebilizumab as a standard maintenance therapy for this patient population.

## Author Contributions

Hongyu Zhou, Mengcui Gui, Jiayang Zhan, and Wei Li performed the conception and design of the study. Hongyu Zhou, Mengcui Gui, Jiayang Zhan, Wei Li, Tao Jin, Dan He, and Bitao Bu participated in study protocol revision and collecting patients' data. Daishi Tian, Zhijun Li, Jing Lin, and Yue Li collected patients' data. Mengcui Gui, Jiayang Zhan, and Wei Li performed the statistical analysis and drafted the manuscript. Hongyu Zhou performed a critical revision of the manuscript. All authors reviewed and approved the final manuscript.

## Funding

The study was supported by Beijing Huikang Charity Foundation (BHCF20240820240808).

## Conflicts of Interest

The authors declare no conflicts of interest.

## Data Availability

The datasets used and/or analyzed during this study are available from the corresponding author upon reasonable request.
